# Middleware Design for Swarm-Driving Robots Accompanying Humans

**DOI:** 10.3390/s17020392

**Published:** 2017-02-17

**Authors:** Min Su Kim, Sang Hyuck Kim, Soon Ju Kang

**Affiliations:** 1 Department of Software Convergence, Kyungpook National University, 80 Daehakro, Bukgu, Daegu 702-701, Korea; totoro0143@gmail.com (M.S.K.); ds_sh@naver.com (S.H.K.); 2 School of Electronics Engineering, College of IT Engineering, Kyungpook National University, 80 Daehakro, Bukgu, Daegu 702-701, Korea

**Keywords:** mobile robot, human-accompanying robot, human-swarm interaction

## Abstract

Research on robots that accompany humans is being continuously studied. The Pet-Bot provides walking-assistance and object-carrying services without any specific controls through interaction between the robot and the human in real time. However, with Pet-Bot, there is a limit to the number of robots a user can use. If this limit is overcome, the Pet-Bot can provide services in more areas. Therefore, in this study, we propose a swarm-driving middleware design adopting the concept of a swarm, which provides effective parallel movement to allow multiple human-accompanying robots to accomplish a common purpose. The functions of middleware divide into three parts: a sequence manager for swarm process, a messaging manager, and a relative-location identification manager. This middleware processes the sequence of swarm-process of robots in the swarm through message exchanging using radio frequency (RF) communication of an IEEE 802.15.4 MAC protocol and manages an infrared (IR) communication module identifying relative location with IR signal strength. The swarm in this study is composed of the master interacting with the user and the slaves having no interaction with the user. This composition is intended to control the overall swarm in synchronization with the user activity, which is difficult to predict. We evaluate the accuracy of the relative-location estimation using IR communication, the response time of the slaves to a change in user activity, and the time to organize a network according to the number of slaves.

## 1. Introduction

The supply and demand of human-supporting robots have been continuously increasing. Therefore, the various methods for this type of robot have been studied, e.g., the vision-based method, the sensor network method, and the wireless-communication strength method. This study focuses on Pet-Bot [1] among the various human-supporting robots.

**Figure 1:**
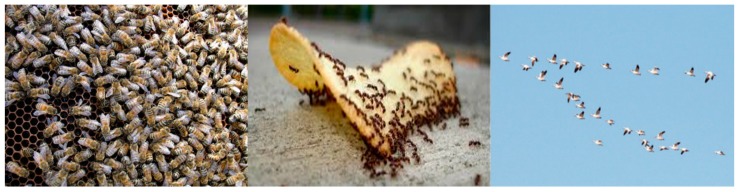
Examples of swarms.

Pet-Bot is a robot that accompanies a human by using LF (Low Frequency) wireless communication to identify a specific user and his/her location. Its purposes are to provide walking-assistance and to carry objects. The Pet-Bot in previous research had a limited number of robots available to each user. This caused problems when a user had to carry many objects at one time or had something too heavy to be carried by a single robot. Swarm is a population whose members have an organic relationship with each other, e.g., bees, ants, and migratory birds, as shown in Figure 1. Moreover, it has two remarkable characteristics: optimal method detection and a parallel process for accomplishing a goal. There has been research that mimics swarm’s characteristics. One study, which mimics swarm’s optimal method detection, is the ACO (Ant Colony Optimization) [2]. However, in this study, since we focus on an extension of Pet-Bot’s application area and utilization, we adapt swarm’s parallel solving process, which is suitable for overcoming the limit of previous Pet-Bots.

In this study, we propose middleware to perform swarm-driving of accompanying robots, focusing on swarm-intelligence robotics to overcome the Pet-Bot’s limit.

To implement the swarm-driving proposed in this study, the relative location and other information must be exchanged between the robots participating in a swarm. Moreover, an independent network must be organized to avoid confusing information between swarms. For these requirements, we use the IEEE 802.15.4 MAC protocol for communication within a swarm, and infrared communication strength for relative-location identification between the participant robots in a swarm. In addition, we use ultrasonic sensors to avoid collisions and maintain a constant distance between robots; an AHRS (Attitude Heading Reference System) is used to control each robot participant’s spin.

The main contribution of this study are as follows.

(i) We adapt the concept of swarm to a human-supporting robot that accompanies the user. This can improve those robots’ application area and utilization.
(ii) The middleware we propose for swarm is composed of a sequence manager, a message-processing manager, and a relative-location identification manger. Swarm-driving is implemented by the cooperation of managers in the middleware. Messages are exchanged between robots through RF communication, and relative-location identification is implemented by IR signal strength.
(iii) For formation control synchronized with the user in real time, we combine a “leader–follower” control method, which has low complexity to implement and is a distribution control method, and a centralized control method, which broadcasts user-activity to all robots in the swarm.
(iv) The performance of this middleware was evaluated. Accuracy of relative-location identification using IR communication was evaluated. Moreover, the response time of the master and slave to user-activity change was measured. In addition, elapsed time to organize network accordance with the number of robots in the swarm was measured.

The rest of this study is organized as follows: Section 2 presents an overview of related work. Section 3 provides a detailed design of our work. Section 4 presents the results of the experiments using our prototype robots. Finally, Section 5 summarizes our contributions and discusses future extensions.

## 2. Related Research

### 2.1. Pet-Bot

**Figure 2:**
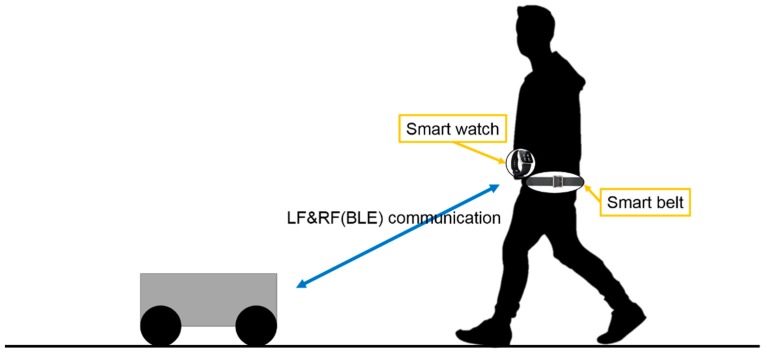
Overall setup of the Pet-Bot system.

The Pet-Bot system provides walking-assistance and helps in carrying objects with a mobile robot that accompanies a user wearing smart wearable devices, as shown in Figure 2. To provide these services, it is essential to identify the authorized user and his/her location. The Pet-Bot uses LF communication, which is used in vehicle smart keys, radio navigation, etc. for this purpose. Authorized user identification is implemented by assigning a unique pattern ID to the LF signal and the smart wearable devices’ LF receivers. An LF signal with the same pattern ID as the LF receiver will wake up a smart wearable device that is in idle state and convert it to active state. Moreover, the Pet-Bot is capable of accurately identifying a specific user’s location using an LF signal having high transmissivity, which allows it to be transmitted through obstacles.

Since the Pet-Bot can provide leading, following, and remote-control modes to suit the user’s requirements, it can provide suitable driving according to the user’s need or the driving environment. However, the Pet-Bot in previous research allowed only a limited number of robots per user. Due to this restriction, it cannot transfer multiple cargoes at a single time or carry a heavy cargo that required more than one robot. Therefore, in this study, we suggest swarm-driving, which allows additional robots to join and cooperate with the user.

### 2.2. Methods for Building Formations

Research on multiple-robot formations have been conducted, focusing on leader–follower, virtual structure, and behavior-based methods.

The leader–follower [3,4,5,6] is a distributed control method in which a specific robot does not manage all the control factors. It assigns a unique name to each robot in the swarm for identification. In this method, the robots are first selected as leaders or followers, and each follower is then made to move to a reference position near its leader. Moreover, this method can effectively swarm-drive multiple robots by dividing them into a hierarchy.

In the virtual structure method [7,8,9], the overall formation of the robots is considered as one fixed body. A master-role robot or external computer computes all the calculations related to the formation control and relays the results to all the robots. Therefore, it knows the next location of each robot. However, this method requires a powerful processor built into the robot, or a computer, since the calculation for estimating the location is highly complex. Any problem in the master-role robot or external computer could cause overall system failure, because of its centralized calculation and formation-control notification.

The behavior-based method [10,11,12] integrates the various goal-oriented behaviors and each robot decides how to accomplish its own goal. In this method, each robot assigns a relatively high priority to behaviors that are more important for accomplishing its goal, and integrates the behaviors according to its priorities. Each robot executes the result of the integrated behavior for its goal. This method is suitable for areas where each robot needs to move autonomously, since its behavior-decision process is very distributed; however, it is difficult to analyze each robot’s behavior mathematically.

The swarm-driving method proposed in this study must drive in sync with the user activity while maintaining the swarm formation. The Pet-Bot synchronizes with user activity that is hard to predict, and it is therefore hard to predict the path for the swarm formation. Therefore, the leader–follower method, which selects a user-connected robot as the leader and makes the other robots build a formation, is suitable for Pet-Bot’s swarm-driving. Further, this method does not require a powerful processor or complex calculations, since it uses a simple criteria to build the formation. To support smoother driving that synchronizes with the user activity, the user-connected robot announces its driving and user-activity information. Therefore, the formation is controlled by the leader–follower method that is combined with a method that periodically shares the master-role robot’s driving and user activity information.

## 3. Detailed Design

### 3.1. Basic Concept

**Figure 3:**
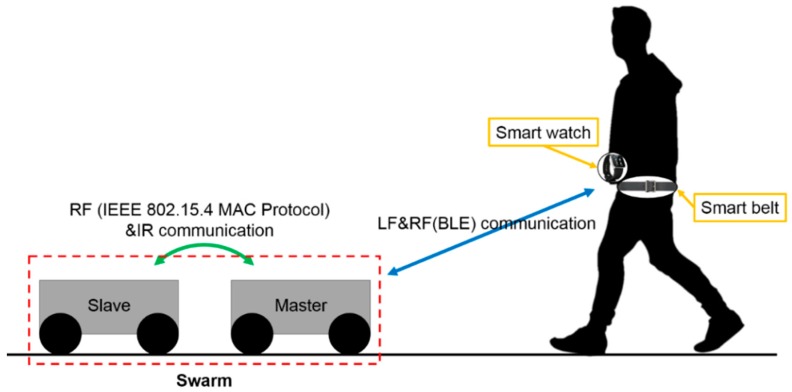
Overall setup of the Pet-Bot system to support swarm-driving.

Figure 3 shows the overall setup of the Pet-Bot system to support swarm-driving. The user wears a smart watch for sending commands to the robot, and a belt for providing location and activity information. The Pet-Bot uses LF communication for user location identification, and BLE (Bluetooth Low Energy) to obtain information of the user location and activity. Using the middleware proposed in this study, the Pet-Bot can organize a swarm of at least two robots, according to the user’s request through the smart watch, as shown in Figure 3. The role of each robot in the swarm is classified as master or slave, according to the state of connection with the user. The robots in the swarm use the RF communication to exchange essential information for building and maintaining the formation, and IR communication, which is suitable for short-distance estimation, for relative-location identification.

**Figure 4:**
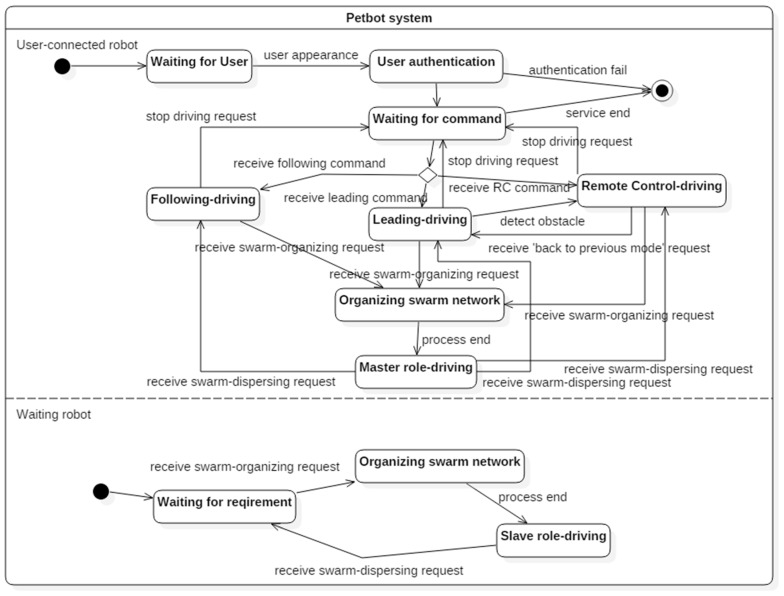
State diagram of the overall Pet-Bot system.

Figure 4 shows the state diagram of the overall Pet-Bot system. The user-connected robot can enter the state for organizing the swarm network from all the supported driving states (leading, following, and remote control), according to the user’s request. Then, the user-connected robot sends the swarm-organizing request to the waiting robots. After the swarm network is organized, the user-connected robot enters the master-role driving state. When the user requests the swarm to disperse, the robot reenters its previous driving state. The waiting robots around the user-connected robot enter the organize-swarm-network state when they receive the swarm-organizing request from the user-connected robot. After the swarm network is organized, the robots enter the slave-role driving state and return to the waiting state when they receive the swarm-dispersing request.

### 3.2. Middleware Architecture for Swarm-Driving

**Figure 5:**
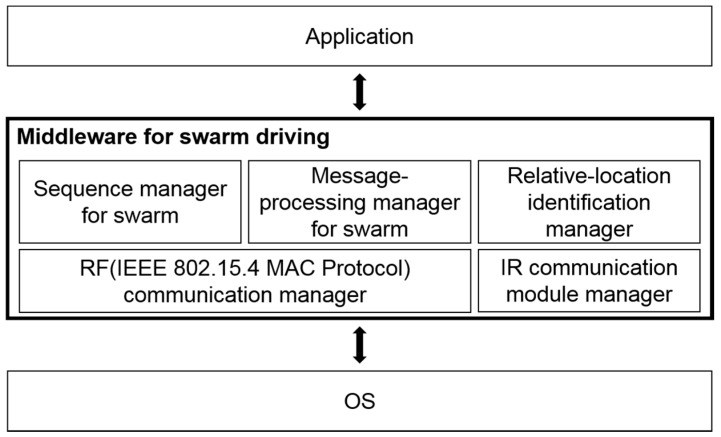
Middleware architecture for swarm-driving.

Figure 5 shows the middleware architecture for swarm-driving. The lower part of the middleware is composed of an RF communication manager and an IR communication module manager managing module’s modes in accordance with the state of the swarm-process. Above them are the sequence manager that manages organizing, maintaining, and dispersing the swarm, the message-processing manager that manages the message exchange of the overall swarm process, and the relative-location recognition manager that helps maintain the formation. The sequence manager progresses overall process according to messages from other robots in the swarm. Then, the sequence manager at specific stage of process configure other managers in the middleware to accomplish the goal of that stage.

### 3.3. Relative-Location Identification

**Figure 6:**
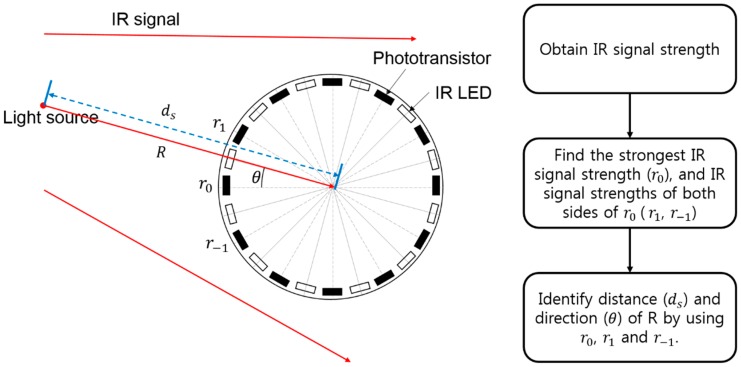
IR communication module’s architecture and parameters, process sequence for relative-location identification.

Figure 6 shows the architecture of the IR communication module and parameters, process sequence for relative-location identification. The transmitters (IR LEDs) and receivers (phototransistors) are set at the top and bottom of the module’s circumference. The module is built at the center of the robot to improve the identification accuracy. The receivers set in the module’s circumference can receive IR signals from all directions, and the transmitters can transmit an IR signal simultaneously in all directions. Since we assigned sequential numbers to each receiver, the module can estimate not only the distance but also the direction of the IR signal source. In this study, the module identifies distance from other robots using IR signal strength following the process sequence in Figure 6. First, the module measures IR signal strength from all directions. Then, find the strongest IR signal strength and strength of both sides of that signal. Finally, module identify distance and direction by using these strengths and specific equations.

(1) $$r_{0}=r*cos\;\theta ,\;r_{1}=r*cos\left(\theta -\frac{\pi }{6}\right),\;r_{-1}=r*cos\left(\theta +\frac{\pi }{6}\right)$$

(2) $$a=\frac{r_{1}+r_{-1}+2r_{0}}{2cos\left(\frac{\pi }{6}\right)+2},\;b=\frac{r_{i}-r_{-i}}{2sin\left(\frac{\pi }{6}\right)}$$

(3) $$\theta =arctan\left(\frac{b}{a}\right),\;r={\left(a^{2}+b^{2}\right)}^{1/2},\;\phi =\frac{n\pi }{6}+\theta$$

Equations (1)–(3) are for the IR signal-strength base relative-location identification; they were modified from [13]. Equation (1) shows the relationship between the measured signal strength and the actual signal strength (R) from a light source; Equations (2) and (3) were developed based on this equation. $r_{0}$ is the strongest signal strength among the measured signal strengths; $r_{1}$ and $r_{-1}$ are the signal strengths on each side of the phototransistor that measured $r_{0}$. When the measured strengths are applied to Equation (2), it estimates the *x* component as *a*, and the *y* component as *b* of the location vector from a light source to the module. With Equation (3), we estimate the distance using the estimated signal strength (*r*) and the direction (θ). If the number of the phototransistor that measured $r_{0}$ is not 0, we estimate the direction (*ϕ*) by adding an additional angle to *θ*.

Each robot needs to know the relative location of the nearby robots to build a formation. To obtain this information, each robot in the swarm must sequentially transmit its IR signal. It is impossible to synchronously transmit or receive the IR signal using each robot’s inner timer because each robot has an independent hardware. To solve this problem, we use a token for the time-synchronized IR communication.

**Figure 7:**
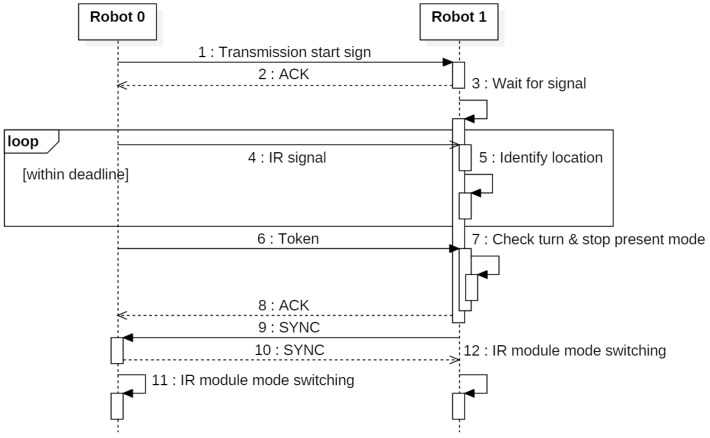
Sequence diagram for sequential IR signal transmission using a token.

Figure 7 shows the sequence of the sequential IR signal transmission using a token. The robot with the token sends the transmission-start sign and waits for response messages. After receiving response messages from the other robots, the robot with the token transmits the IR signal until the deadline. When the deadline is reached, the robot with the token broadcasts the token message, including the number of the robot with the next turn, and waits for the response messages. After receiving the response messages, the robot with the token waits for sync messages from the other robots. After the sync-message switching, the robot with the number within the token message switches its IR module to transmission mode, and the other robots switch their IR modules to reception mode. This process is iterated until the end of the swarm-driving. Through this token method, we can guarantee time-synchronized sequential IR communication.

### 3.4. Swarm-Network Formation

The swarm-network formation process forms an independent network based on a waiting robot’s response to the joining query, and the number of robots required by the user.

Table 1 shows the criterion for a waiting robot to decide whether to join. This criterion is composed of a connection with a user and the distance from the master. By assigning a higher priority to condition of connection with a user, the distance-checking process can be omitted if the robot is already connected with any user. The robot which is not connected with any user and is located within the swarm organizing available region of the master, sends a joining-available message to the master; a robot that does not satisfy the joining criterion sends a joining-unavailable message to the master.

Table 2 shows the criterion for deciding which robots will join a master. In the first case, if the user-required number is smaller than the number of available robots, the master selects the robots that are closest to it. In the second case, if the user-required number is greater than or equal to the number of available robots, the master selects all of the robots.

**Figure 8:**
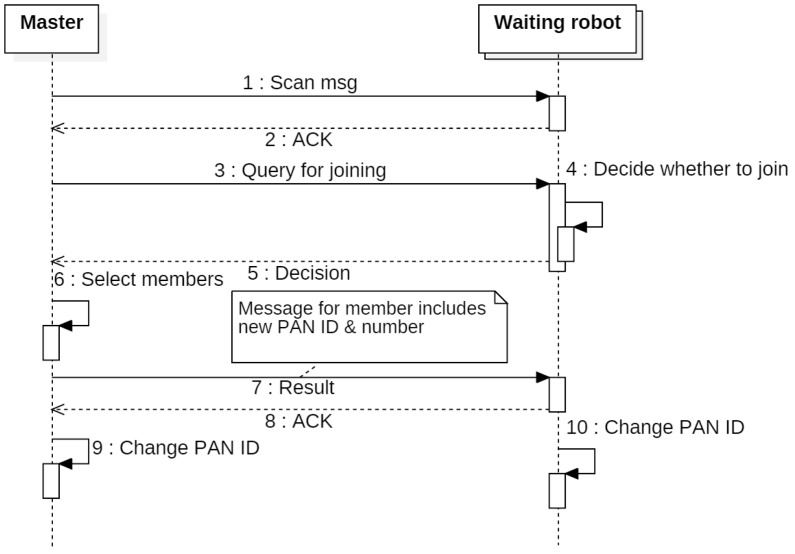
Sequence diagram of the process for organizing a swarm network.

Figure 8 shows the sequence diagram of the process for organizing a swarm network. A master broadcasts a scan message to search for waiting robots and estimates the number of joining-candidate robots based on the responses to the scan message. Then the master broadcasts the joining query to the joining-candidate robots. Waiting robots that receive the joining-query decide their joining intention through the criteria, and send that decision to the master. The master makes the joining-candidate list and selects the joining robots through the criteria. It then sends the results of its decision. If the robots receive a non-participating notification, they enter the waiting state. If the robots receive a joining notification, the robots form an independent network by changing their PAN (Personal Area Network) ID to the new PAN ID included in the notification message. In addition, each robot can identify its neighbor robots by using the total number of swarm members and the number assigned to each robot in the notification message.

### 3.5. Swarm Formation and Maintenance

In this study, we used the leader–follower-based swarm-formation control method to move the swarm synchronously with the user. Since the leader–follower method uses only information of distance and direction from the leader robot to build the formation, followers can easily build the formation, and move in accordance with the user-activity change while keeping the formation. The user-connected robot sends its speed and user-activity changes to all robots in the swarm, to sensitively react to the user’s speed or direction changes. Moreover, since the robots align the direction of the swarm through the heading information obtained by IR communication, each robot exchanges the heading information with its neighbor robots. To maximize the degree of freedom of the swarm-formation area, each robot acquires relative-location information from at least its neighbor robots and exchanges heading information. The swarm formation varies depending on how the user selects each robot’s neighbor robots according to the intended use.

**Figure 9:**
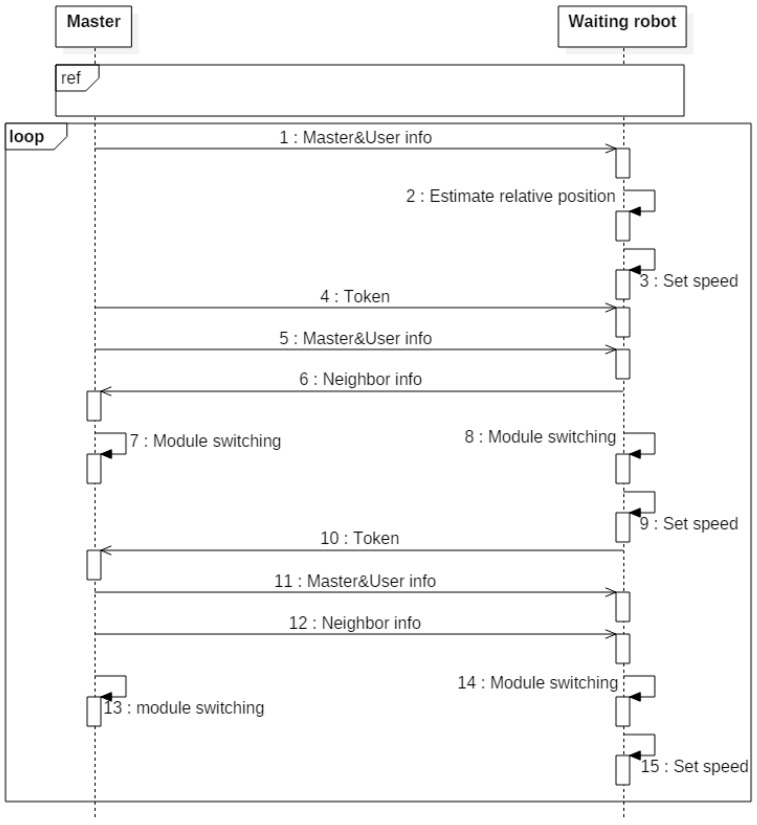
Simple swarm-driving process.

Figure 9 shows the simple swarm-driving process. After organizing the swarm network, the master periodically sends information on its speed and user-activity changes to the slaves. The neighbor robots of the robot transmitting the IR signal send heading information to the transmitting robot every IR-communication role-changing period. Each slave decides its speed and direction using environmental information obtained by its sensors and by comparing the heading information received from its neighbors and the latest relative-location information obtained from each robot.

**Figure 10:**
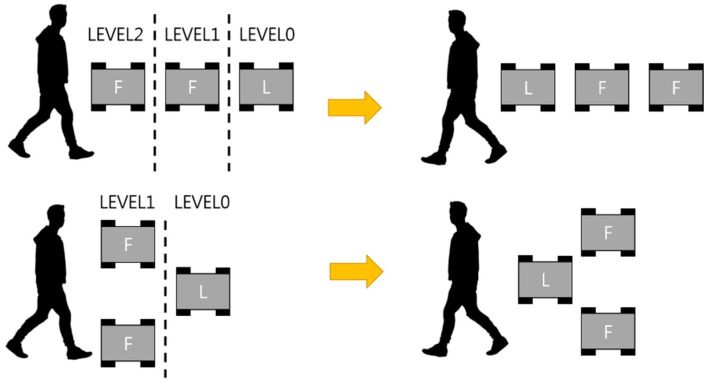
Examples of master change and formation change.

Moreover, the control method designates the master-role robot according to the driving mode and the shape of the formation, for a more natural swarm movement. In both leading and following modes, the user-connected robot is selected as the master. However, the difference between leading and following modes is the master’s location from the user. In following mode, the master is located closest to the user, but in leading mode the master is located farthest from the user, as shown in Figure 10. The master location in leading mode can make the master lead the other robots smoothly because it is less influenced by the location identification and the communication delay between the robots. Therefore, in leading mode, the formation building is started after the master is preferentially shifted, according to the hierarchical level shown in Figure 10. This hierarchical levels are assigned a lower number at the farthest robot in sequence. The master finds the need to move to the designated location, or figures out where to move, using this hierarchy.

Moving the position of the master and building the formation accordingly minimizes the time delay that occurs when a new master is connected to user and the swarm-network is reorganized, which occurs in the case when the formation is created first. When the driving mode is changed, as shown in Figure 10, the master change and the change of the slaves’ relative location are decided by the criteria in Table 3. Following Table 3, if Master-change request is ‘No change’ condition and driving-mode change occurs, the master is not changed, but the neighbor robot’s relative-location reference is. Moreover, if Master-change request is the ‘Change’ condition and driving-mode change occurs, the master is changed, but the neighbor robot’s relative-location reference is not.

### 3.6. Main Controllers for Each Role

#### 3.6.1. Control Factors for the Main Controllers

**Figure 11:**
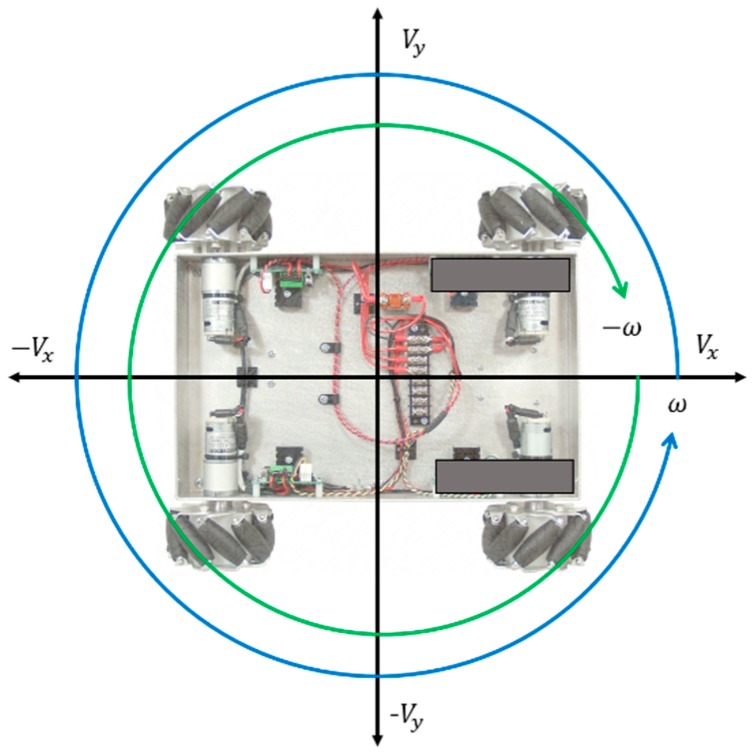
Pet-Bot control factors.

Figure 11 presents the control factors for the main controllers. The control factors are composed of $V_{x}$ (front and rear speed), $V_{y}$ (left and right speed), and ω (angular speed). All robots in a swarm accomplish their goals by calculating these control factors through their main controllers.

#### 3.6.2. Master-Role Main Controller’s Goal and Architecture

The master-role main controller’s goal is to keep the robot a standard distance (1 m) and direction from the user. To accomplish this goal, the master-role controller calculates the control factors based on user location, activity, and driving service. See [1] for details.

#### 3.6.3. Slave-Role Main Controller’s Goal and Architecture

**Figure 12:**
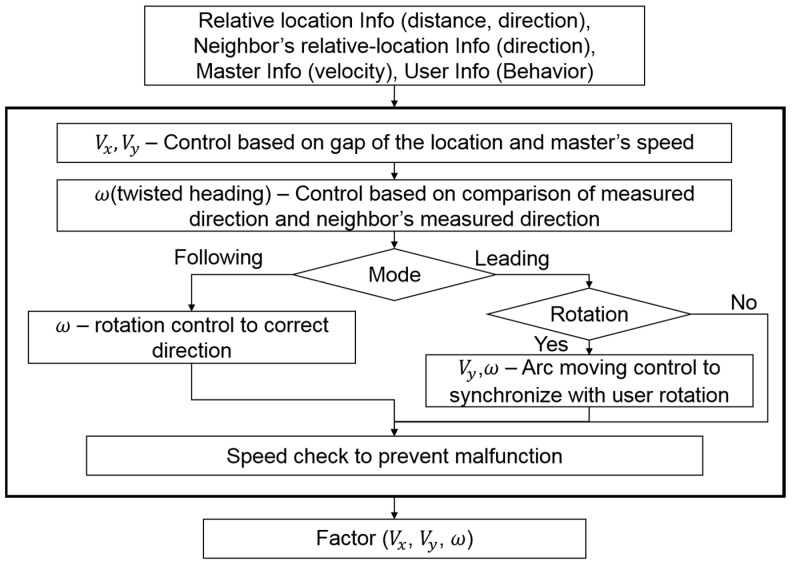
Flow chart of slave-role main controller’s control-factor configuration process.

The slave-role main controller’s goal is to have a robot move to a predicted location to build a formation and maintain its distance and direction from the neighbor robots. To accomplish this goal, the slave-role controller uses the relative location, its neighbor robot’s relative location, the master’s driving speed, and the user activity, as shown in Figure 12.

**Figure 13:**
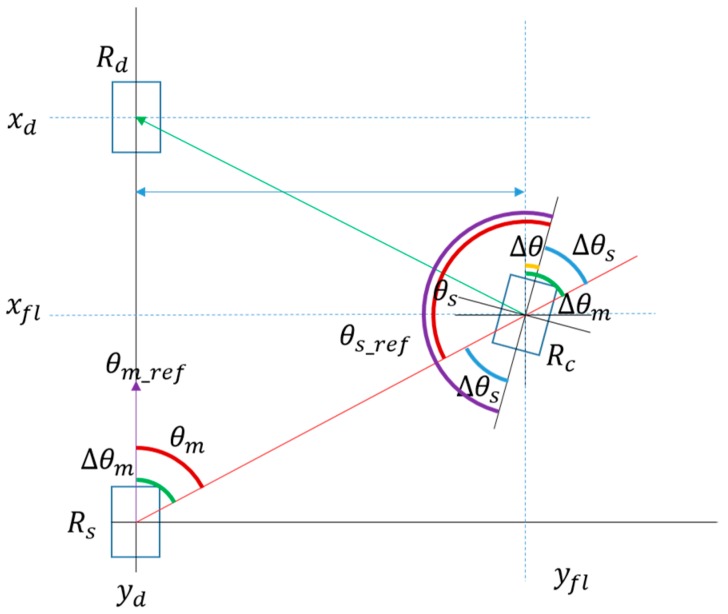
Geometry of a robot’s twisted-degree estimation for direction alignment.

The slave-role main controller calculates $V_{x}$ and $V_{y}$ based on the result of comparing the reference location with the actual location. $V_{x}$ and $V_{y}$ are revised based on the master’s driving-speed information. This process is needed for better synchronization with the user activity and to compensate for the input-data update delay caused by the sequential IR communication process. Then, all robots in a swarm must correct their twisted heading to align with the swarm’s heading. In this study, we did not use a magnetic compass, which is easily affected by the flux of a magnetic field. Instead, we used the direction part of each robot’s estimated relative location to estimate its degree of twisted heading. After the robot’s main controller estimates its degree of twisted heading, it selects a moving method for aligning the robot with the user according to the rotational motion of the user. When the robot detects the user rotation, in leading mode, the robot will move along the arc, and in following mode, the robot just rotates toward the correct direction. See [1] for details. Figure 13 shows the method for estimating the twisted heading. $R_{c}$ is the actual location of the robot. $R_{d}$ is the reference location of the robot. $\Delta \theta_{m}$ is the angle gap between $\theta_{m\_ref}$ and $\theta_{m}$, and $\Delta \theta_{s}$ is the angle gap between $\theta_{s\_ref}$ and $\theta_{s}$. Δθ is the angle gap between these two angles; this is the robot’s twisted-heading level. The slave-role main controller corrects the twisted angle by adjusting the angular speed (ω) until ∆*θ* is within the error range.

Two methods are used for calculating the control factors related to the user’s rotation, according to the robot’s driving mode. In the leading mode, all robots in a swarm must do arc driving. To do this, the master, which can obtain user-rotation information in real time, periodically sends this information to all robots in the swarm. Then, each robot except the master must change $V_{y}$ or ω considering its distance from the user. In the following mode, a robot does not have any specific calculation method for user rotation; it simply keeps moving toward the reference location. Through this process, robots in a swarm can maintain their formation, and synchronize with the user activity when they are in the driving state.

## 4. Implementation and Evaluation

### 4.1. Hardware Configuration

**Figure 14:**
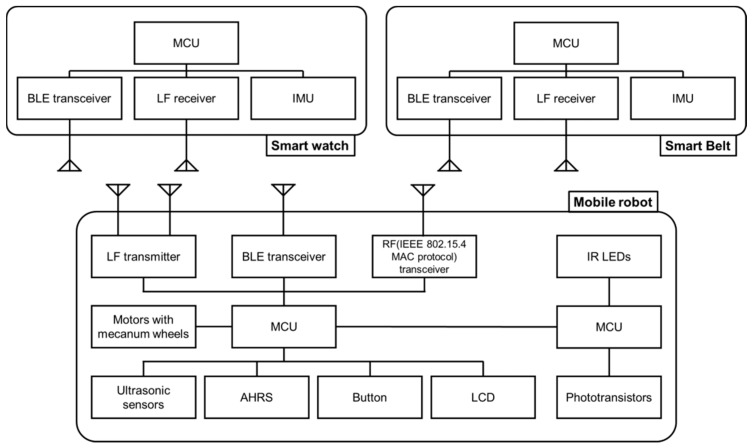
Pet-Bot system’s hardware configuration.

Figure 14 shows the overall hardware configuration of the swarm-driving Pet-Bot system. This system is composed of a mobile robot and two smart wearable devices. The smart wearable devices consist of an ARM Cortex-M3-based MCU (microcontroller) (1024 KB flash memory, 128 KB RAM), a BLE transceiver for exchanging information with a robot, an LF receiver to identify an authorized user and his/her location, and an IMU (Inertial Measurement Unit) to recognize user activity and gesture commands.

A mobile robot consists of an ARM Cortex-M4-based MCU (1024 KB flash memory, 128 KB SRAM, floating-point calculation), a BLE transceiver, LF antennas, and an RF (IEEE 802.15.4 protocol) transceiver for exchanging information between robots in a swarm. It also includes an AHRS for obtaining the robot’s posture information, motors, mecanum wheels, ultrasonic sensors, buttons, and an LCD. An IR communication module, composed of an ARM Cortex-M3-based MCU, IR LEDs, and phototransistors, is loaded onto the robot.

The wearable devices, mobile robot’s main board, and IR communication module use the real-time ‘Ubinos’ operating system, which supports multitasking functions, since all components of the Pet-Bot system must respond to changes in the environment.

**Figure 15:**
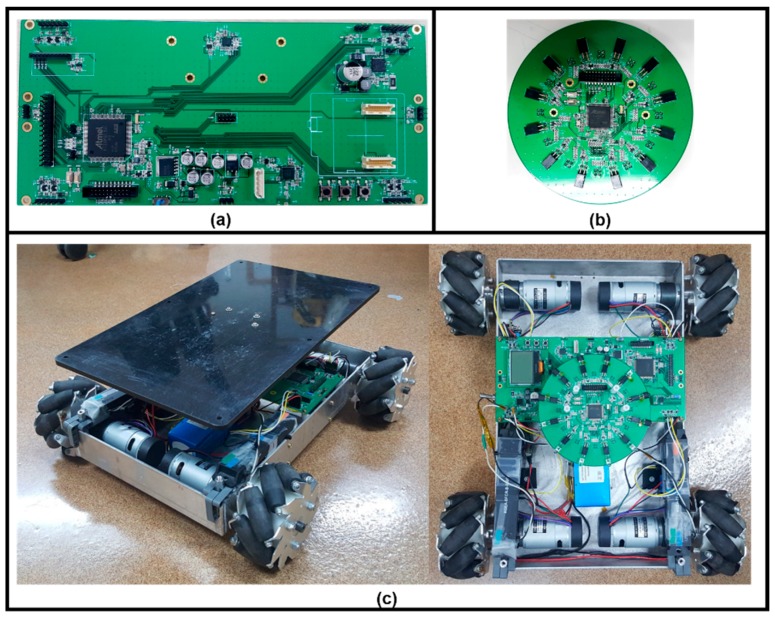
Physical prototypes: (**a**) main board; (**b**) IR communication module; and (**c**) mobile robot.

Figure 15 shows prototypes of a main board, an IR communication module, and a mobile robot. We conducted evaluation tests using these prototypes.

### 4.2. Software Architectures

**Figure 16:**
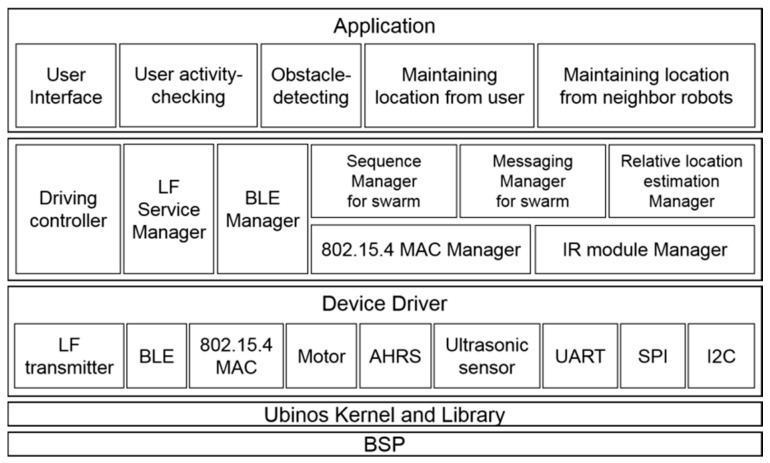
Software architecture of main board.

**Figure 17:**
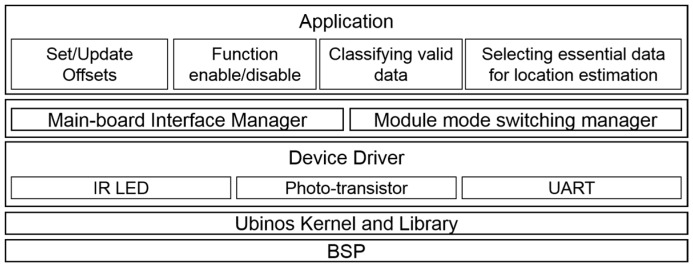
Software architecture of infrared communication module.

Figure 16 and Figure 17 show the main board’s software architecture and the IR communication module’s software architecture, respectively. Since both of them use the real-time Ubinos OS, the kernel and libraries for Ubinos are present in the board support package. Moreover, there are device drivers for peripherals to support Pet-Bot’s services. Middleware for managing the peripherals is located above the device drivers. The main board contains middleware for swarm-driving in this layer. Finally, applications for providing Pet-Bot’s overall services are located on the top layer of the software architecture.

### 4.3. Evaluation of Relative-Location Identification Accuracy

**Figure 18:**
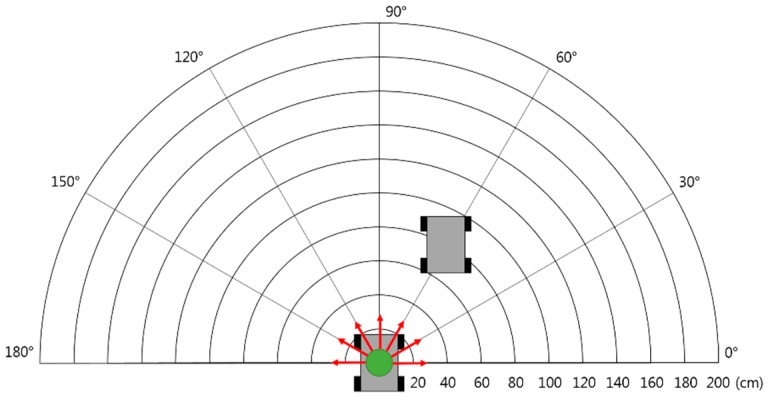
Environment for accuracy evaluation of relative-location estimation using IR communication.

This evaluation is to verify the accuracy of the relative-location identification method using IR communication. We place the robot that transmits the IR signal in the center; the robot that receives the IR signal is located by changing the distance and angle, as shown in Figure 18. The distance varied from 20 cm to 40 cm in a range from 40 cm to 200 cm, and the angle varied from 0° to 180° in 30° increments. The receiving robot measured the IR signal 300 times at each point.

**Figure 19:**
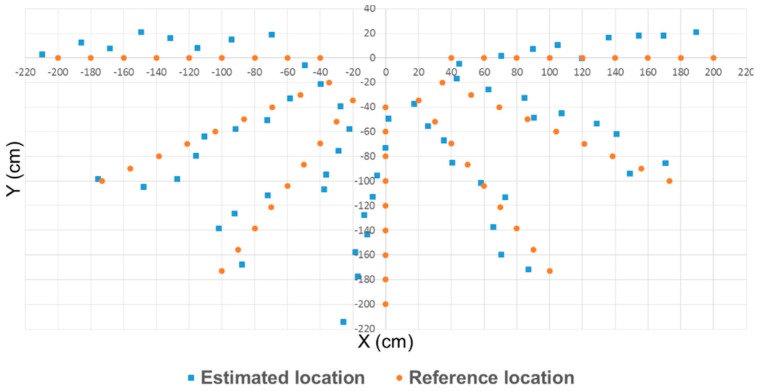
Result of relative-location recognition using IR signal intensity.

Figure 19 shows the result of the receiving robot’s relative-location identification as a two-dimensional plot. In this plot, the circle means the actual location of the IR-signal-receiving robot and the square means the estimated location.

Table 4 shows the root mean square error (RMSE) values of the distance and direction. The results were a 7.898 cm RMSE and 6.52° RMSE. In this study, since each robot has to be located only within a certain range of the reference location, the result of the relative-location identification is considered sufficient for use as the input for the main controller.

### 4.4. Reaction Time to User Activity Change

The middleware architecture suggested in this study is designed to broadcast user activity information from the master to all slaves in a swarm. A time delay inevitably occurs in this process. In order to verify whether this delay would cause problems in swarm-driving, we measured the reaction time to user activity change 50 times. The reaction time is defined as the interval from the start point of the user rotation to the start point of the slave’s motion.

**Figure 20:**
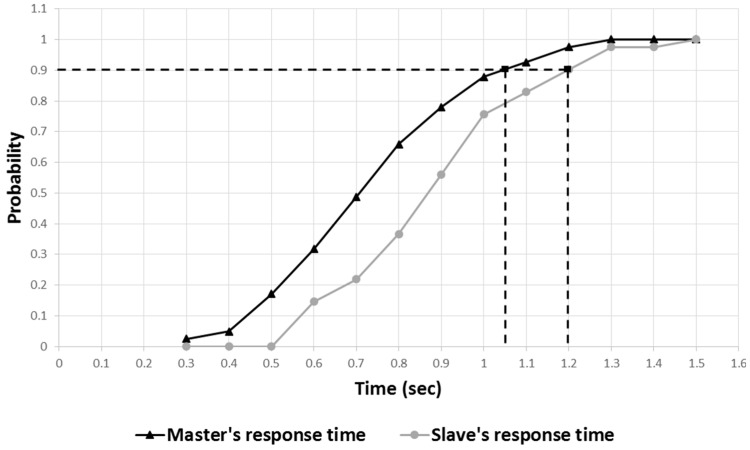
Cumulative distribution of master and slave’s reaction time to user activity change.

**Figure 21:**
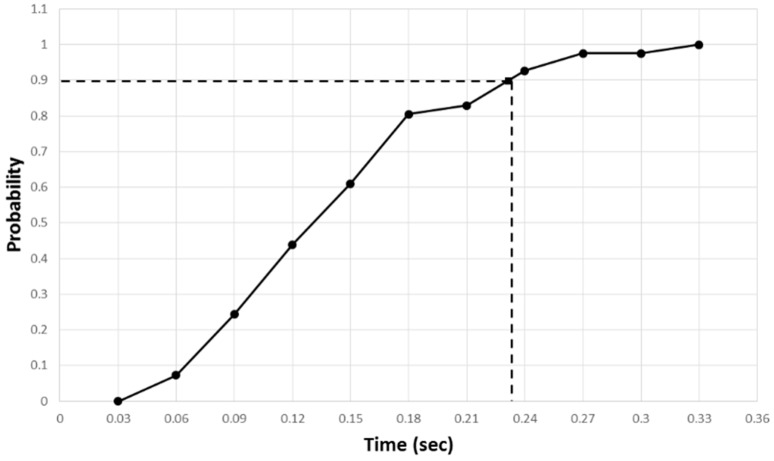
Cumulative distribution of reaction time interval between master and slave.

Figure 20 shows the cumulative distribution of the master and slave’s reaction time to user activity change. The evaluation result showed a 90% probability that the master’s reaction time would be within 1.1 s and a 90% probability that the slave’s reaction time would be within 1.2 s. Figure 21 shows that the slave responded within 0.24 s with a 90% probability, based on the user activity information from the robot. Through repeated evaluations, we empirically confirmed that this time interval was sufficient for the slave to drive in synchronization with the user activity. Therefore, the communication-time delay between the master and the slave needed to synchronize with the user activity, is acceptable for achieving the goal proposed in the study.

### 4.5. Comparison of Time for Organizing a Network, According to the Number of Slaves

The middleware suggested in this study must be able to organize a swarm network according to the user-required number of slaves. The middleware includes a retry process in case the network cannot be organized, at a time due to simultaneous message reception or message corruption.

To compare the network-organizing time according to the number of slaves, the evaluation included varying the number of slaves from one to five, organizing each network 50 times, and measuring the time.

**Figure 22:**
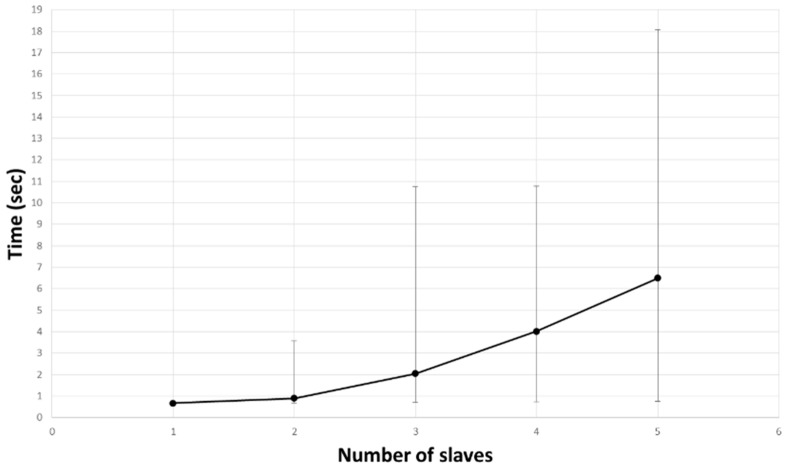
Elapsed time to organize a network, according to the number of slaves.

Figure 22 shows the average times for organizing a swarm network according to the number of slaves. As the number of slaves increases, the average network-formation time tends to increase. The error bar at each point represents the minimum required time and the maximum required time. The minimum required time is the case for organizing the swarm without retrying. This time increases slightly from 0.6 s to 0.8 s, depending on the number of slaves. The middleware retries when it determines that not all of the response messages corresponding to the request message have arrived; thus, the maximum required time is determined according to the number of times.

It can be seen that factors other than the number of slaves influence the maximum required time, since the maximum required time is the nearly same when the number of slaves is three and four.

Table 5 shows the retry probability according to the number of slaves. As the number of slaves increases, the retry probability increases. This is due to a failure to receive the entire message, caused by the increase in the number of simultaneously arriving messages as the number of slaves increases.

## 5. Conclusions

In this study, we described a study that was based on the Pet-Bot, a human-supporting robot with a limit to the amount that can be used. To extend various application areas and utilization by overcoming that limit, we adapted the concept of the swarm to the Pet-Bot. Since this concept can effectively achieve such a common goal the swarm wanted, it is suitable for overcoming that limit. Therefore, we propose the middleware for swarm-driving the accompanying robot. This middleware is divided into three major managers; process sequence, message-processing, and relative-location identification managers. The process sequence manager manages the sequence of the overall process of the swarm-network and driving. The message processing manager is in charge of message communication within the independent swarm-network using RF communication. The relative-location identification manager enables all robots to identify their neighbors’ relative location by using IR communication. To synchronize with the user activity more smoothly, we use the formation control method that is combined with the leader–follower method, which is a distributed and centralized method. In the evaluation with prototypes, we confirmed that the accuracy of relative-location identification is sufficient to be used in swarm-driving, and the reaction time to user activity change is short enough to reflect the user activity in real time. Moreover, we confirmed that the average elapsed time to organize the swarm-network has an increasing tendency following an increased number of slaves due to retry. As a result, we expect the Pet-Bot to be applicable at more various areas that require carriage services. In the future, we need to proceed to improve the accuracy and acquisition time of relative-location to make the swarm more smoothly synchronized to user activity. Additionally, we should decrease the number of retries in the organizing swarm-network.

## Figures and Tables

**Table 1:** Criterion for a waiting robot to decide whether to join.

Region	Inside of Available Region	Outside of Available Region
Connection		
**Not connected**	Can connect	Cannot connect
**Connected with other users**	Cannot connect	Cannot connect

**Table 2:** Criterion for deciding which robots will join a master.

Case	Result
Number of user-required robots < number of robots that can join	Select the robots which are close to the master
Number of user-required robots ≥ number of robots that can join	Select all robots which can join

**Table 3:** Criteria for changing the master when the driving mode is changed.

Master-Change Request	Change	No Change
Driving-Mode Change		
Leading → Following	Select the closest robot as the master, but do not change the neighbor robots’ relative-location reference	Do not change the master, but change the neighbor robots’ relative-location reference
Following → Leading	Select the robot with the highest hierarchical level as master, and the neighbor robots’ relative-location reference	Do not change the master, but change the neighbor robots’ relative-location reference

**Table 4:** RMSE of distance and direction.

RMSE	
Distance (cm)	7.898
Direction (°)	5.8

**Table 5:** Retry probability per number of slaves.

Number of Slaves	1	2	3	4	5
**Retry Probability (%)**	0	6	30	67	80
